# How to Give Mercury

**Published:** 1901-09-07

**Authors:** 


### HOW TO GIVE MERCURY.

We have no doubt that the great majority of practitioners habitually give mercury by the mouth in cases where they require the specific action of this drug, as, for example, in the treatment of syphilis, and probably in the great majority of cases they get satisfactory results. At any rate they may do if their patients will pay proper attention to diet, to the teeth, and to the intestinal tract generally. At the same time cases are met with by no means infrequently in which the results so obtained are not the best possible, and in such the choice often lies between the old plan of inunction, and the more recently-introduced method of deep intra-muscular injection. Dr. C. F. Marshall[1] has lately been comparing the advantages and disadvantages of these two methods. The injection method consisted of the injection of 10 minims of a solution of sal alembroth (containing one-third of a grain of the salt) deeply into the muscles of the buttock by means of a platinum-iridium needle. The inunction was done with unguentum cinereum, consisting of one part of mercury, one part of lanolin, and half a part of olive oil About half a drachm of this preparation was rubbed daily into different parts of the body, the rubbing lasting 20 minutes. A warm bath was taken each time before the inunction. From observation of a considerable number of cases which were under treatment in hospital for a year or more, Dr. Marshall concludes that relapses are twice as frequent with the injection treatment as with the inunction method, and in this view he says he is supported by observations on several hundreds of cases which were watched for shorter periods. The great advantages claimed for injection are secrecy, cleanliness, uniformity of dose, and that it is performed by the surgeon himself. Its disadvantage is the pain, but it is said that injections of sozoiodol of mercury or of Lang's grey oil are less painful than those of the above-mentioned mixture. The great disadvantages of inunction are irksomeness and dirtiness. It would seem, however, to be the more reliable method of the two, and the best to adopt where rapid mercurialisation is required, as in cases of iritis.

[1] Lancet, Aug. 24.

